# A study on EEG differences between active counting and focused breathing tasks for more sensitive detection of consciousness

**DOI:** 10.3389/fnins.2024.1341986

**Published:** 2024-03-12

**Authors:** Yimeng You, Yahui Li, Baobao Yu, Ankai Ying, Huilin Zhou, Guokun Zuo, Jialin Xu

**Affiliations:** ^1^Cixi Biomedical Research Institute, Wenzhou Medical University, Ningbo, Zhejiang, China; ^2^Ningbo Institute of Materials Technology and Engineering, Chinese Academy of Sciences, Ningbo, Zhejiang, China; ^3^Ningbo Cixi Institute of Biomedical Engineering, Ningbo, Zhejiang, China; ^4^University of Chinese Academy of Sciences, Beijing, China

**Keywords:** consciousness detection, disorders of consciousness, event-related potential, electroencephalogram, machine learning

## Abstract

**Introduction:**

In studies on consciousness detection for patients with disorders of consciousness, difference comparison of EEG responses based on active and passive task modes is difficult to sensitively detect patients’ consciousness, while a single potential analysis of EEG responses cannot comprehensively and accurately determine patients’ consciousness status. Therefore, in this paper, we designed a new consciousness detection paradigm based on a multi-stage cognitive task that could induce a series of event-related potentials and ERD/ERS phenomena reflecting different consciousness contents. A simple and direct task of paying attention to breathing was designed, and a comprehensive evaluation of consciousness level was conducted using multi-feature joint analysis.

**Methods:**

We recorded the EEG responses of 20 healthy subjects in three modes and reported the consciousness-related mean event-related potential amplitude, ERD/ERS phenomena, and the classification accuracy, sensitivity, and specificity of the EEG responses under different conditions.

**Results:**

The results showed that the EEG responses of the subjects under different conditions were significantly different in the time domain and time-frequency domain. Compared with the passive mode, the amplitudes of the event-related potentials in the breathing mode were further reduced, and the theta-ERS and alpha-ERD phenomena in the frontal region were further weakened. The breathing mode showed greater distinguishability from the active mode in machine learning-based classification.

**Discussion:**

By analyzing multiple features of EEG responses in different modes and stimuli, it is expected to achieve more sensitive and accurate consciousness detection. This study can provide a new idea for the design of consciousness detection methods.

## 1 Introduction

Patients with severe disorders of consciousness (DOC) are those who have impaired perception of their surroundings or themselves due to severe brain injury, cerebral hemorrhage or infarction, electrocution, cardiac disease, drowning, etc. The consciousness state of patients with DOC is classified as unresponsive wakefulness syndrome (UWS)/vegetative state (*VS*) and minimally conscious state (MCS) (Giacino et al., 2014).

UWS/*VS* patients retain basic brainstem reflexes and the sleep–wake cycle, they are able to open their eyes spontaneously or upon stimulation but lack awareness of their environment (Laureys et al., 2010). MCS patients have weak and discontinuous consciousness and own a certain degree of behavioral response (Giacino et al., 2002). The current clinical misdiagnosis rate of patients with DOC is as high as about 40% (Schnakers et al., 2009). After patients are misdiagnosed, the lack of timely and correct treatment may lead to further deterioration of the condition and even death. Therefore, accurate assessment of consciousness state is essential for clinical rehabilitation therapy of patients with DOC (Demertzi et al., 2008).

In order to reduce the clinical misdiagnosis rate, many new methods have been proposed for consciousness detection to assess the state of consciousness (Gosseries et al., 2014; Marino et al., 2016; Kondziella et al., 2020). Among them, electroencephalography (EEG) examination based on event-related potentials (ERPs) is increasingly used for the diagnosis and assessment of clinical disorders of consciousness, which has the advantages of high temporal resolution and relatively low cost (Górska and Binder, 2019). In clinical medicine, consciousness refers to the brain’s ability to perceive, recognize and respond to the surrounding environment and its own state (Sarasso et al., 2014). Cognitive potentials reflect the neurophysiological changes in the brain during the cognitive process (Vanhaudenhuyse et al., 2008). They are able to assess the content of consciousness, such as perception, attention, and memory (Sculthorpe-Petley et al., 2015), and thus detect consciousness. However, due to the variability of brain injury and the fluctuating level of consciousness in patients with DOC, it is often necessary to collect multiple evoked potentials with different latencies during cognitive processes in the brain several times in order to determine the patient’s level of consciousness (Sergent et al., 2017). The conscious state of a healthy person is stable, and the EEG responses to external stimuli are highly reproducible. The first step in applying consciousness detection methods to patients with DOC is to accurately and sensitively detect consciousness in healthy persons (Höller et al., 2013a,b; Morlet et al., 2017, 2023). The validity of the experimental paradigm and potential components representing different consciousness contents needs to be validated in healthy persons (Cruse et al., 2014). Therefore most studies on consciousness detection have set up healthy controls (Soddu et al., 2009).

Detecting the presence or absence of ERP components associated with consciousness is the basic method of consciousness detection. These components are P300, CNV, N400, P600, etc. In the studies of consciousness detection based on P300, auditory P300 was induced by auditory stimuli such as calling the patient’s name or calling a stranger’s name (Kempny et al., 2018), and visual P300 was induced by visual stimuli such as presenting a picture of a target digit and a picture of a non-target digit (Pan et al., 2014) or haptic P300 was induced by the vibration of a stimulator placed at different locations on one’s limb (Murovec et al., 2020). In these studies, it was found that the patients who possessed the P300 component tended to be among the MCS patients. Some of the *VS* patients with detectable P300 components had improved consciousness over time. Contingent negative variation (CNV) is a classical cognitive potential that manifests itself as a negative potential change in the frontal cortex, reflecting the subject’s anticipation of a commanded stimulus (Macar and Vidal, 2004). Studies showed that CNV can be observed in the EEG responses of MCS patients and *VS* patients. Still, Patients with CNV tended to notice abnormalities in the stimulation pattern, and P300 components were more likely to exist in their EEG responses (Rozier et al., 2020). In addition, Schoenle and Witzke’s study suggested that N400 could be used to distinguish between non-UWS, near-UWS, and UWS (Schoenle and Witzke, 2004). In Wu’s study, they found that the P600 was absent in UWS patients, so that in combination with the P600, the distinction between MCS and UWS could be achieved. In these ERP-based consciousness detection studies, calling the subject’s name and presenting a photograph of the subject were often used as auditory stimuli and visual stimuli. This is because these self-relevant stimuli can put the subject in a better cognitive state and induce more pronounced ERPs (Laureys et al., 2007).

The task modes of the consciousness detection paradigm are divided into two main categories based on the demands placed on the subject: the active mode and the passive mode (Risetti et al., 2013). The active mode requires the subject to focus on a specific stimulus and perform the associated mental task, and the passive mode places no demands on the subject, who is not required to respond to the stimulus. Using only one of these modes, it is often impossible to determine whether a subject’s EEG response to a stimulus is automatic or voluntary, and thus the patient’s state of consciousness cannot be accurately determined. Comparing EEG responses in the two modes is a more reliable way of determining the state of consciousness, for example, conscious people have higher P300 amplitudes in the active mode than in the passive mode. It is worth noting that the ERP component is not entirely absent in the passive mode compared to the active mode, even if the subject is not asked to respond to the stimulus after receiving the stimulation (Escera et al., 1998). This is because in the passive mode, conscious subjects constantly change their attention to targets, and their attention will be easily attracted to stimuli such as calling names or presenting photographs. ERPs such as the P300 are induced to some extent, which will reduce the difference from that in the active mode. Thus, comparing the EEG responses of active and passive modes is not an optimal method of consciousness detection. In 1989, Polich showed that setting a secondary task was able to reduce the amplitudes of ERPs in subjects compared to an ignoring condition (Polich, 1989). In a related study on consciousness detection, Morlet et al. (2017) proposed to amplify the EEG difference between the mental imagery mode and the active mode by imagining hanging out at home, thus improving the sensitivity of consciousness detection. This method was validated for feasibility on healthy persons. On the other hand, patients who possessed consciousness without behavioral responses had been shown to be able to perform mental imagery tasks, such as playing soccer, golf, etc., to achieve simple communication (Owen et al., 2006; Cruse et al., 2011). Still, as mental imagery tasks were often complex and challenging, they were only applicable to some DOC patients with a high degree of consciousness.

In addition, in recent years, many machine-learning based consciousness detection methods have been proposed. Pan et al. (2020) designed a variety of brain-computer interfaces for consciousness detection, and the accuracy was obtained by classifying the EEG responses based on support vector machines (SVM), and there was a significant correlation between the accuracy and consciousness recovery. Altintop et al. extracted EEG features such as entropy, Hjorth parameter, complexity, etc. and used algorithms such as Multilayer Perceptron Neural Networks and Random Forests to classify the level of consciousness (Altintop et al., 2022, 2023). Meanwhile, algorithms such as the synthetic minority oversampling technique (SMOTE) and the spatio-temporal self-constructing graph neural network (ST-SCGNN) also provide solutions to the data imbalance and cross-subject classification in consciousness detection research (Chawla et al., 2002; Pan et al., 2023). Consciousness detection through machine learning is a possible approach.

In this study, we aimed to improve the sensitivity and accuracy of consciousness detection by exploring appropriate paradigms and electrophysiological features. We proposed a simple and straightforward breathing mode, which could be applied to most patients with DOC. Under the breathing mode, the subject’s brain reduced the processing of external stimuli, and the related EEG responses were significantly weakened or even disappears, which was compared with the EEG responses under the active mode to determine whether the subject responded to the task actively or not. By this method, it was expected that sensitive detection consciousness would be achieved. Meanwhile, compared with the traditional oddball unimodal paradigm, we designed a multi-stage cognitive task under visual and auditory conditions, which could induce ERP components related to different consciousness contents. And through the joint analysis of multi-potential features, the accuracy of consciousness detection improved. Unlike most studies that focused only on the analysis of ERPs and ignored the non-phase-locked information, we further analyzed the spectral oscillation distribution features of the brain to assist consciousness detection (Harrison and Connolly, 2013). Components such as P300 and N400 in ERPs and the event-related desynchronization/synchronization(ERD/ERS) phenomena of the EEG rhythm, as the neurophysiological features of the brain related to consciousness, could reflect consciousness contents such as semantic processing, attentional engagement and so on. Based on the paradigm designed and proposed electrophysiological features in this paper, quantifying EEG differences under different conditions using machine learning is expected to be used for consciousness detection.

## 2 Materials and methods

### 2.1 Participants

Data were collected from 20 healthy subjects (13 males and 7 females, mean age of 24.2 ± 1.3 years). Their native language was Chinese. Exclusion criteria were hearing deficits, visual deficits, neurological or psychiatric history, and taking of sedative treatment. The experiment was approved by the Bioethics Committee at Ningbo Institute of Materials Technology and Engineering, Chinese Academy of Sciences. Before the experiment, all subjects were informed of the experimental procedure and signed an informed consent form.

### 2.2 Experimental procedure

**Figure 1:**
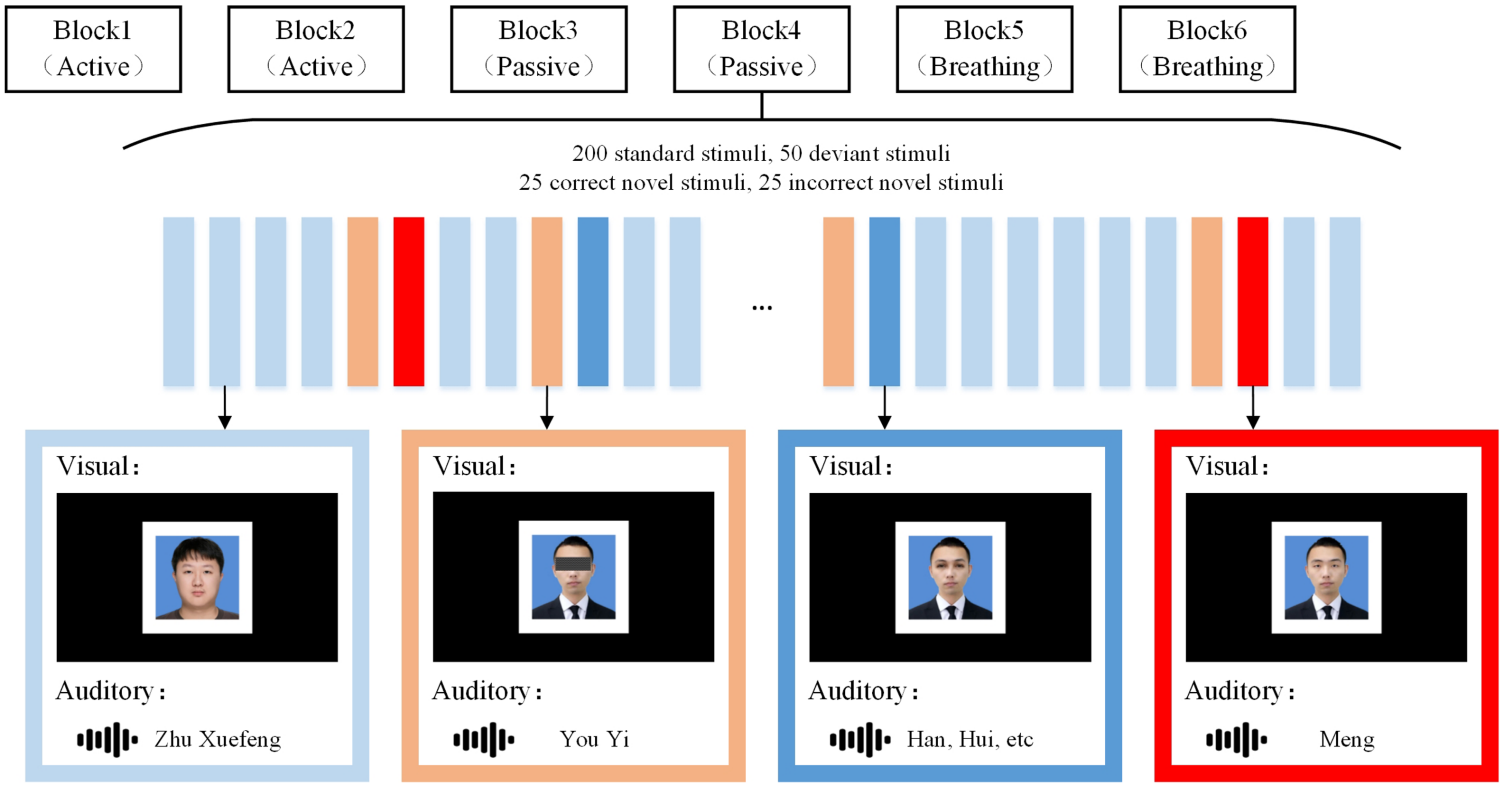
Stimulus paradigm. Under the visual and auditory paradigms, corresponding standard stimuli, deviant stimuli, correct novelty stimli, and incorrect novelty stimuli were presented. Under each sensory stimulus, the active mode, the passive mode, and the breathing mode each occupied two blocks, and the subject was given a request at the beginning of each block. Here, the subject’s name was “You Yimeng” and the stranger’s name was “Zhu Xuefeng.” Instructions and stimuli were presented through a monitor and headphones.

Our experiments contained visual and auditory paradigms in which subjects’ EEG was recorded in three task modes (1) active mode, focus on the target stimulus and count. (2) Passive mode, nothing is required. (3) Breathing mode: pay attention to breathing and ignore stimuli. All paradigms contained four types of stimuli: the standard stimulus, the deviant stimulus, the correct novel stimulus (the target stimulus), and the incorrect novel stimulus. The specific flow of the experiment is shown in Figure 1.

In the visual paradigm, the standard stimulus was a photograph of a stranger with an unobstructed full face. The deviant stimulus was a photograph of a subject with occluded eyes, the correct novel stimulus was a photograph of a subject with occlusion removed, and the incorrect novel stimulus was a photograph of a non-subject removing occlusion and changing the facial feature of the eyes. All photographs were presented in white boxes, with borders designed to make subjects more clear where the photographs appeared. In the auditory paradigm, the standard stimulus was to call out the stranger’s name, the deviant stimulus was to call out the first half of the subject’s name, such as “You Yi” for “You Yimeng.” The correct novel stimulus was to call out the last word of the subject’s name, such as “Meng” for “You Yimeng,” whereas the incorrect novel stimulus was to call out an irrelevant alternative word, such as “Hui,” “Han,” and so on. Standard stimuli, deviant stimuli, correct novel stimuli, and incorrect novel stimuli all lasted for 600 ms, with deviant stimuli separated from novel stimuli by 600 ms, and the rest of the stimuli were randomly spaced at intervals ranging from 600 to 1,000 ms. Each paradigm contained 6 blocks, and each block contained 200 standard stimuli, 50 deviant stimuli, 25 correct novel stimuli and 25 incorrect novel stimuli. Before the start of each block, subjects were given the appropriate instructions, with the active mode prompting “Count the correct photo/name,” the passive mode prompting “Accept stimuli but do not respond,” and the breathing mode prompting “Ignore stimuli and focus on your breathing.” Blocks were presented in a particular order, with a 5-min break between each block. Auditory stimuli were recorded by a male native Chinese speaker with guaranteed duration, and all audio was loudness-matched. Picture processing software was used to ensure that the photographs in the visual stimuli were of the same size. During EEG recording, stimuli were presented via Eprime 2.0. All experimental tasks were performed in an electromagnetically shielded room to minimize environmental interference.

Based on the above experimental procedure, it was promising to induce a series of potentials in different spatial brain regions with different latencies to comprehensively assess the subjects’ consciousness. Experiments were conducted in the active mode, the passive mode, and the breathing mode to verify the differences among the different modes and the advantages of the breathing mode over the passive mode. The visual paradigm and auditory paradigm were also designed to be able to accommodate clinical subjects with auditory or visual impairments due to complications.

### 2.3 Data acquisition and preprocessing

The EEG data were recorded using a Neuroscan EEG system with a sampling rate of 1,000 Hz. The electrode caps were made of 32 Ag/AgCl electrodes, and the electrode distribution followed the international 10/20 system. Two unipolar electrodes were placed on the right and left mastoids (M1, M2). Two pairs of bipolar electrodes were used to record the vertical and horizontal electrooculography (EOG) in order to detect eye movements and blinks.

The EEG data were processed offline using EEGLAB (Delorme and Makeig, 2004). The raw data were band-pass filtered from 0.1 to 20 Hz with a trap filter at 50 Hz and re-referenced based on the average of M1 and M2. After this, counting from the moment when the stimulus was given, the EEG data from 200 ms before this moment to 1,000 ms after this moment were considered as the data segment for this stimulus. Baseline corrections were made using the first 200 ms of data. Eye movements with other artifacts were removed using independent component correlation algorithm (Makeig et al., 1997), in which trials with voltages exceeding ±75 uV were excluded.

### 2.4 ERP analysis

For each subject’s EEG responses to different types of stimuli, we intercepted the EEG data with a duration of 1,200 ms for averaging. As consciousness is closely related to the frontoparietal lobe of the brain, we chose two specific regions of interest (ROI) for the study of EEG characteristics under stimulation: the parietal region (ROI1: P7, P3, PZ, P4, P8), and the frontal region (ROI2: FP1, FP2, F7, F3, FZ, F4, F8). Based on the mean ERP waveform and mean brain topography, we defined P300 amplitude as the average amplitude of ROI1 under deviant stimuli within the time of 250–400 ms, CNV amplitude as the average amplitude of ROI2 under deviant stimuli within the time of 800–1,000 ms. N400 amplitude was the difference between the mean amplitude of ROI1 under incorrect novel stimuli and correct novel stimuli in 380–420 ms. P600 amplitude was the difference between the mean amplitude of ROI2 under incorrect novel stimuli and correct novel stimuli in 650–750 ms.

### 2.5 ERD/ERS analysis

Time-frequency decomposition was performed using the short-time Fourier transform to obtain the power spectrum of the EEG signals compared to the baseline. Event-related spectral perturbation (ERSP) analysis was performed under a fixed 200 ms Hanning window (Grandchamp and Delorme, 2011). For each stimulus, data were taken 200 ms before the stimulus moment and 1,000 ms after the stimulus at a frequency of 0.1–20 Hz for calculation. The calculation method is as follows:


$$P\left(t,\;f\right)={\left|F\left(t,\;f\right)\right|}^{2}$$

For each trial, the baseline was set from −200 to 0 ms. Baseline correction was achieved by the subtraction method in the time-frequency domain:


$$P_{bl}\left(t,f\right)=P\left(t,f\right)-\bar{R}\left(f\right)$$

Where $\bar{R}\left(f\right)$ is the average power spectral density of the baseline interval at each frequency. $P_{bl}\left(t,\;f\right)$<0 is considered to represent ERD, and $P_{bl}\left(t,\;f\right)$>0 is considered to represent ERS.

We chose to analyze the time-frequency characteristics of the FZ electrodes, the average ERD/ERS power values in the theta (4–7 Hz) 200–400 ms after stimulation and in the alpha (8–13 Hz) 400–1,000 ms after stimulation were selected for analysis.

### 2.6 Classification

In order to quantify the degree of differences in EEG response across modes and stimuli, we used SVM to calculate the accuracies of EEG responses classification between standard and deviant stimuli or between correct novelty stimuli and incorrect novelty stimuli in different modes, as well as the accuracies of EEG responses classification under a specific type of stimulus between active and passive modes or between active and breathing modes (Zhao et al., 2022). SVM implemented in Matlab software through fitcsvm function, the kernel function of SVM is linear, and the parameter ‘OptimizeHyperparameters’ is set to ‘auto’ to optimize the hyperparameters. Meanwhile, we used the SMOTE to solve the problem of data imbalance in standard and deviant stimulus classification. The EEG data were downsampled to 100 Hz. We extracted 1,000 ms of EEG data from all leads after stimulation and concatenated them as features. In the EEG classification under standard and deviant stimuli, deviant stimuli were labeled as +1 and standard stimuli were labeled as −1. In the EEG classification under novel stimuli, correct novel stimuli were labeled as +1, and incorrect novel stimuli were labeled as −1. In the EEG classification under active and other modes, active modes were labeled as +1, and other modes were labeled as −1. We calculated and averaged the 20 subjects’ classification accuracy, sensitivity, and specificity.

When the number of trials averaged was one, half of all the samples are randomly taken as the training set and the other half as the test set to train the model and calculate the classification accuracy. The operation was repeated 10 times to obtain the average classification accuracy. In addition, we also used the EEG data after a certain number of trials averaged as the test set to calculate the classification accuracy, and recorded the change of classification accuracy during the number of trials averaged from one to five.

### 2.7 Statistical analysis

First, *a priori* analysis of the required sample size was performed using G-Power. The sample size of 20 for two-tailed paired *t*-tests was estimated for an alpha error probability of 0.05, a power of 0.8, and an effect size of 0.67. The sample size of 60 for one-way analysis of variance (ANOVA) was estimated for an alpha error probability of 0.05, a power of 0.8, and an effect size of 0.42. The sample size for each group was 20.

Statistical analysis software SPSS 25 was used for further analysis. The results are expressed as mean ± SE. We used paired *t*-tests to test for significant differences in the following data: (1) potential amplitudes under standard and deviant stimuli; (2) Potential amplitudes under correct and incorrect novel stimuli; (3) The accuracies of active mode EEG in distinguishing from passive mode as well as from breathing mode. For the following data we used a one-way ANOVA to test the effect of mode: (1) Event-related potential potential amplitude in the three modes; (2) ERD/ERS power in the three modes; (3) Classification accuracy in the three modes. In *post hoc* analyses, pairwise comparisons were performed using the Bonferroni method. Among them, a value of *p*<0.05 was considered statistically significant.

## 3 Results

### 3.1 ERP results

**Figure 2:**
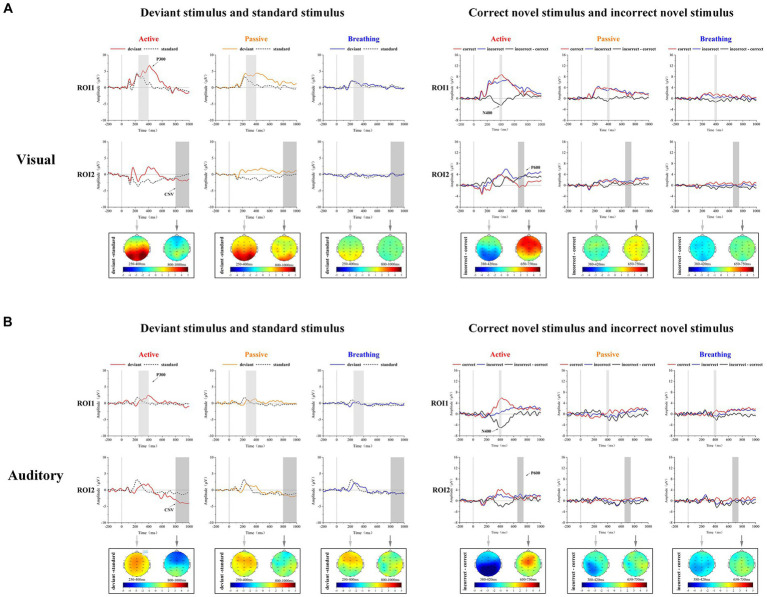
Mean ERP waveforms and brain topographic maps in visual and auditory paradigms for 20 subjects. Panel **(A)** shows the subjects’ EEG responses to standard stimuli and deviant stimuli. The light gray (250–400 ms) and dark gray regions (800–1,000 ms) represent the intervals used for brain topographic mapping and statistical analysis. Panel **(B)** shows the subjects’ EEG responses to correct novel stimuli (red) and incorrect novel stimuli (blue), as well as the difference waveforms between the two (black). The light gray (380–420 ms) and dark gray regions (650–750 ms) represent the intervals used for brain topographic mapping and statistical analysis.

As shown in Table 1, the mean amplitudes of EEG responses under deviant and standard stimuli were statistically analyzed in the visual and auditory paradigms. It was found that the deviant stimulus in the visual paradigm could induce stronger positive ERP components than the standard stimulus in active and passive modes, and the deviant stimulus in the auditory paradigm induced stronger positive ERP components than the standard stimulus in active mode. As shown in Figure 2, a significant P300 component appeared in the EEG responses to deviant stimuli in these modes. In addition, we found that in the active mode of the auditory paradigm, the deviant stimulus evoked a more significant negative ERP component at 800–1,000 ms than the standard stimulus. In the active mode of the visual paradigm, a negative ERP was also present, but it was not statistically significant. This negative ERP component was not observed in the passive and breathing modes of both paradigms. This implies that the CNV potential is significantly characterized only in the active mode.

**Table 1:** Paired *t*-test results of ERP amplitude under deviant and standard stimuli.

Paradim	Mode	Post-stimulation 250–400 ms time period			Post-stimulation 800–1,000 ms time period		
		Deviant stimulus amplitude (μV)	Standard stimulus amplitude (μV)	*p*-value	Deviant stimulus amplitude (μV)	Standard stimulus amplitude (μV)	*p*-value
Visual	Active	4.63 ± 0.60	2.32 ± 0.46	***p* < 0.001**	−1.72 ± 0.67	−0.35 ± 0.36	*p* = 0.060
	Passive	3.84 ± 0.84	1.61 ± 0.42	***p* = 0.001**	0.83 ± 0.38	−0.12 ± 0.25	***p* = 0.033**
	Breathing	1.31 ± 0.43	1.06 ± 0.35	*p* = 0.380	−0.11 ± 0.31	0.03 ± 0.20	*p* = 0.692
Auditory	Active	1.35 ± 0.27	0.57 ± 0.20	***p* = 0.013**	−3.82 ± 0.47	−1.07 ± 0.23	***p* < 0.001**
	Passive	0.95 ± 0.44	0.32 ± 0.20	*p* = 0.163	−1.76 ± 0.92	−1.23 ± 0.26	*p* = 0.525
	Breathing	0.03 ± 0.39	0.04 ± 0.15	*p* = 0.979	−0.98 ± 0.90	−0.96 ± 0.20	*p* = 0.983

*p*-values < 0.05 are bolded.

As shown in Table 2, the mean amplitudes of EEG responses to correct and incorrect novel stimuli were statistically analyzed in both the visual and auditory paradigms. It was found that the incorrect novel stimulus induced more negative potential components at 380–420 ms than the correct novel stimulus in all modes except the passive mode. As shown in Figure 2, a clear N400 can be observed in the difference waves in these modes. In the active mode of the visual paradigm, the incorrect novel stimulus evoked a larger positive ERP component at the 650–750 ms time period, which was not observed in the other modes. Thus, we observed a significant P600 component in the difference wave only in the active mode of the visual paradigm.

**Table 2:** Results of paired *t*-tests for ERP amplitude with correct and incorrect novel stimuli.

Paradim	Mode	Post-stimulation 380–420 ms time period			Post-stimulation 650–750 ms time period		
		Correct novel stimulus amplitude(μV)	Incorrect novel stimulus amplitude(μV)	*p*-value	Correct novel stimulus amplitude (μV)	Incorrect novel stimulus amplitude(μV)	*p*-value
Visual	Active	8.63 ± 1.00	6.25 ± 0.85	***p* < 0.001**	−0.32 ± 0.88	2.86 ± 1.07	***p* < 0.001**
	Passive	3.78 ± 1.01	3.04 ± 0.73	*p* = 0.283	0.60 ± 0.66	1.51 ± 0.96	*p* = 0.106
	Breathing	1.57 ± 0.56	0.35 ± 0.65	***p* = 0.012**	0.24 ± 0.42	−0.06 ± 0.57	*p* = 0.533
Auditory	Active	5.43 ± 0.56	0.52 ± 0.36	***p* < 0.001**	1.02 ± 0.69	1.92 ± 0.53	*p* = 0.240
	Passive	0.60 ± 0.62	−0.96 ± 0.39	*p* = 0.093	0.61 ± 0.77	0.10 ± 0.60	*p* = 0.658
	Breathing	1.08 ± 0.47	−0.63 ± 0.49	***p* < 0.001**	0.45 ± 0.64	0.21 ± 0.66	*p* = 0.748

*p*-values < 0.05 are bolded.

In order to determine the effect of the factor of mode on the amplitude of these potentials, the amplitudes of P300, CNV, N400, and P600 were tested using a one-way analysis of variance (ANOVA), and the results are shown in Table 3. It was found that in the visual paradigm P300 amplitude, CNV amplitude, and P600 amplitude differed significantly between modes. In the auditory paradigm P300 amplitude, CNV amplitude, and N400 amplitude were found to be significantly different between modes. Except for the CNV amplitude in the visual paradigm, in all other cases, the breathing mode was able to differ more from the active mode compared to the passive mode.

**Table 3:** Results of one-way ANOVA and *post-hoc* analysis of ERP amplitude.

Paradim	ERP	One-way ANOVA	*Post-hoc* analysis: active and passive modes	*Post-hoc* analysis: active and breathing modes
Visual	P300	**F(2, 57) = 7.17, *p* = 0.002, *η***^**2**^ **= 0.20**	*p* = 1.000	***p* = 0.002**
	CNV	**F(2, 57) = 7.27, *p* = 0.002, *η***^**2**^ **= 0.20**	***p* = 0.001**	*p* = 0.063
	N400	F(2, 57) = 2.46, *p* = 0.094, *η*^2^ = 0.08	*p* = 0.099	*p* = 0.470
	P600	**F(2, 57) = 11.70, *p* < 0.001, *η***^**2**^ **= 0.29**	***p* = 0.009**	***p* < 0.001**
Auditory	P300	**F(2, 57) = 3.28, *p* = 0.045, *η***^**2**^ **= 0.10**	*p* = 1.000	***p* = 0.047**
	CNV	**F(2, 57) = 3.41, *p* = 0.040, *η***^**2**^ **= 0.10**	*p* = 0.222	***p* = 0.042**
	N400	**F(2, 57) = 8.64, *p* = 0.001, *η***^**2**^ **= 0.23**	***p* = 0.002**	***p* = 0.002**
	P600	F(2, 57) = 0.64, *p* = 0.532, *η*^2^ = 0.02	*p* = 0.869	*p* = 1.000

P1 represents the results of the *post-hoc* analysis of the active and passive modes, P2 represents the results of the *post-hoc* analysis of the active and breathing modes. *p*-values < 0.05 are bolded.

Through the analysis, it was known that in the active mode, there were significant differences in the EEG responses evoked by different types of stimuli and significant ERP components could be observed. Secondly, in the passive mode, there were differences in the part of the EEG responses evoked by different types of stimuli. In the breathing mode, the differences in the EEG responses evoked by different types of stimuli were smaller. Meanwhile, the differences in the amplitude of each ERP component were compared between active and passive modes, as well as the active and breathing modes. It was found that the difference in ERP amplitudes between breathing and active modes was greater than the difference in ERP amplitudes between active and passive modes.

### 3.2 ERD/ERS results

**Figure 3:**
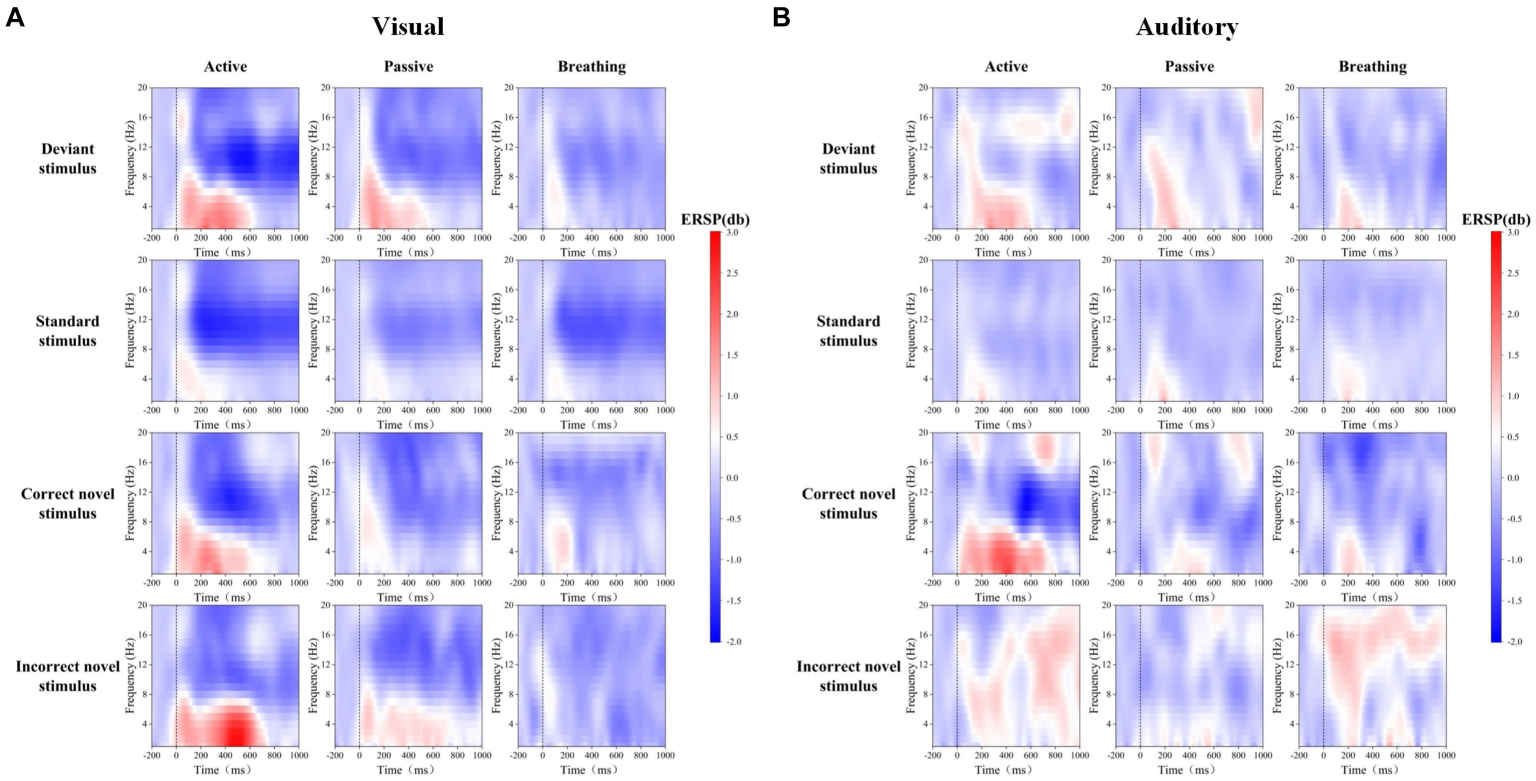
Mean ERSP time-frequency maps of FZ electrodes for 20 subjects in visual and auditory paradigms. Panel **(A)** shows the EEG spectral changes evoked by deviant stimuli, standard stimuli, and novel stimuli in different modes in the visual paradigm. Panel **(B)** shows the EEG spectral changes evoked by deviant stimuli, standard stimuli, and novel stimuli in different modes in the auditory paradigm.

**Figure 4:**
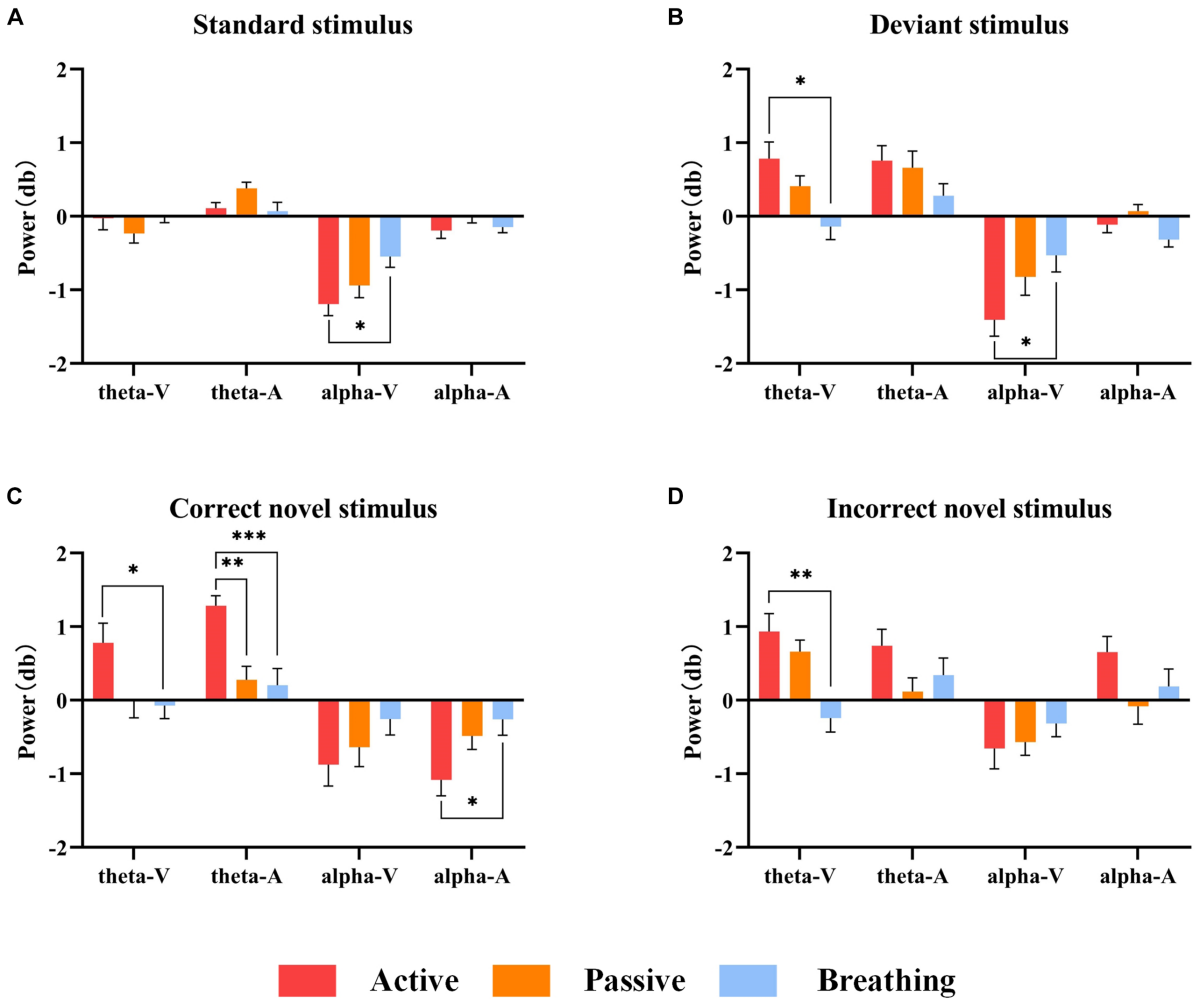
Mean power values of 20 subjects in the theta and alpha frequency bands under different stimuli. Panel **(A)** shows the magnitude of ERD/ERS power under the standard stimulus. Panel **(B)** shows the ERD/ERS power magnitude under the deviant stimulus. Panel **(C)** shows the ERD/ERS power magnitude under the correct novel stimulus. Panel **(D)** shows the ERD/ERS power magnitude under the incorrect novel stimulus. In this figure, ‘*’ denotes *p*  <  0.05, ‘**’ denotes *p* <  0.01, ‘***’ denotes *p* < 0.001.

Frontal zone electrode FZ was selected for time-frequency analysis. As one of the four brain regions, the frontal lobe has functions such as attention regulation, task decision-making, and emotion regulation, which are closely linked to consciousness. Through Figure 3, we can observe the differences in ERD/ERS phenomena under different stimuli. In order to analyze the differences in EEG spectral power between passive and active modes, as well as breathing and active modes, the average power of EEG in the theta frequency band, 200–400 ms, and in the alpha frequency band, 400–1,000 ms, under different stimuli were statistically analyzed, and the results of the time-frequency analysis are shown in Table 4 and Figure 4.

**Table 4:** Results of one-way ANOVA and *post-hoc* analyses of ERS/ERD power.

Paradim	ERS/ERD	Stimulation	One-way ANOVA	P1	P2
Visual	theta-ERS	Standard stimulus	F(2, 57) = 0.98, *p* = 0.381, *η*^**2**^ = 0.03	*p* = 0.755	*p* = 1.000
		Deviant stimulus	**F(2, 57) = 6.23, *p* = 0.003, *η***^2^ **= 0.18**	*p* = 0.483	***p* = 0.003**
		Correct novel stimulus	**F(2, 57) = 4.36, *p* = 0.017, *η***^2^ **= 0.13**	*p* = 0.052	***p* = 0.031**
		incorrect novel stimulus	**F(2, 57) = 9.46, p < 0.001, *η***^2^ **= 0.25**	*p* = 1.000	***p* < 0.001**
	alpha-ERD	Standard stimulus	**F(2, 57) = 4.27, *p* = 0.018, *η***^2^ **= 0.13**	*p* = 0.781	***p* = 0.016**
		Deviant stimulus	**F(2, 57) = 3.62, *p* = 0.033, *η***^2^ **= 0.11**	*p* = 0.255	***p* = 0.031**
		Correct novel stimulus	F(2, 57) = 1.46, *p* = 0.241, *η*^**2**^ = 0.05	*p* = 1.000	*p* = 0.287
		incorrect novel stimulus	F(2, 57) = 0.67, *p* = 0.517, *η*^**2**^ = 0.02	*p* = 1.000	*p* = 0.809
Auditory	theta-ERS	Standard stimulus	F(2, 57) = 3.10, *p* = 0.052, *η*^**2**^ = 0.10	*p* = 0.152	*p* = 1.000
		Deviant stimulus	F(2, 57) = 1.61, *p* = 0.209, *η*^**2**^ = 0.05	p = 1.000	*p* = 0.287
		Correct novel stimulus	**F(2, 57) = 10.35, *p* < 0.001, *η***^2^ **= 0.26**	***p* = 0.001**	***p* < 0.001**
		incorrect novel stimulus	F(2, 57) = 2.15, *p* = 0.126, *η*^**2**^ = 0.07	*p* = 0.136	*p* = 0.581
	alpha-ERD	Standard stimulus	F(2, 57) = 1.11, *p* = 0.338, *η*^2^ = 0.04	*p* = 0.474	*p* = 1.000
		Deviant stimulus	**F(2, 57) = 3.83, *p* = 0.028, *η***^2^ **= 0.12**	*p* = 0.567	*p* = 0.468
		Correct novel stimulus	**F(2, 57) = 4.27, *p* = 0.019, *η***^2^ **= 0.13**	*p* = 0.133	***p* = 0.019**
		incorrect novel stimulus	F(2, 57) = 2.62, *p* = 0.081, *η*^**2**^ = 0.08	*p* = 0.092	*p* = 0.475

P1 represents the results of the *post-hoc* analysis of the active and passive modes, P2 represents the results of the *post-hoc* analysis of the active and breathing modes. *p*-values < 0.05 are bolded.

As a result of the one-way ANOVA, we found that in most cases there were significant differences in ERD/ERS between the different modes. After a post-hoc analysis, it can be seen that there is no significant difference between the active and passive modes in all cases, except for the theta-ERS of the active and passive modes under the correct novel stimuli of the auditory paradigm. On the other hand, the breathing mode showed more variability from the active mode under a larger number of stimuli. It was also found that there were more time-frequency domain differences between modes under the correct novel stimuli compared to other stimuli, not only in theta-ERS but also in alpha-ERD.

In summary, there are different degrees of ERD/ERS phenomena in the time-frequency domain of EEG under different stimuli in the active mode, and because these phenomena differ slightly from those in the passive mode, we cannot judge the subject’s consciousness participation based on these features only. And when we detect the difference in time-frequency domain features between active and breathing modes, we can hopefully judge the existence of the subject’s consciousness.

### 3.3 Classification results

**Figure 5:**
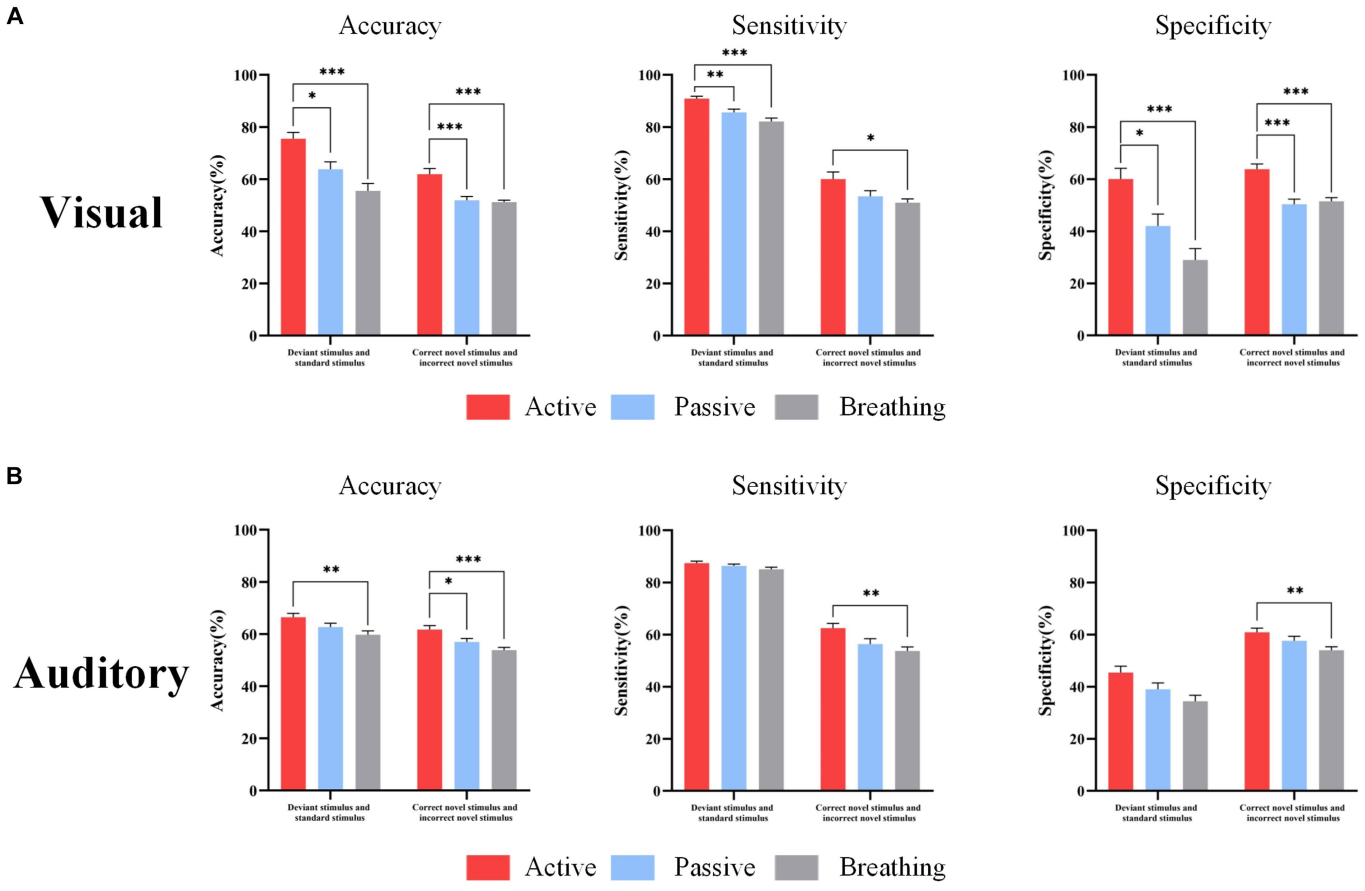
EEG response classification performance under different types of stimuli. Panel **(A)** shows the accuracy, sensitivity, and specificity of distinguishing standard and deviant stimuli, correct novelty and incorrect novelty stimuli in three modes under the visual paradigm. Panel **(B)** shows the accuracy, sensitivity, and specificity of distinguishing standard and deviant stimuli, correct novelty and incorrect novelty stimuli in the three modes under the auditory paradigm. In this figure, ‘*’ denotes *p* <  0.05, ‘**’ denotes *p* <  0.01, ‘***’ denotes *p* < 0.001.

The study classified the EEG of each subject under different types of stimuli in active, passive, and breathing modes by SVM, and calculated the accuracy, sensitivity, and specificity. The specific results are shown in Table 5. The accuracy of distinguishing the EEG response under deviant stimuli from standard stimuli in the active mode reached 75.5 ± 2.5% in the visual paradigm (sensitivity: 90.9 ± 0.8%, specificity: 60.1 ± 4.1%), and 66.5 ± 1.5% in the auditory paradigm (sensitivity: 87.5 ± 0.7%, specificity 45.5 ± 2.4%). It can be concluded that these two types of EEGs in the active mode are distinguishable and the characteristic differences are significant. The accuracy of distinguishing the two types of EEGs in the passive and breathing modes was lower than that in the active mode, where there was a large difference in the classification accuracy between the breathing mode and the active mode. The accuracy was only 55.6% in the breathing mode of the visual paradigm and 59.8% in the breathing mode of the auditory paradigm. Second, in the active mode, the accuracy of distinguishing EEG responses under correct novel stimuli from incorrect novel stimuli reached 62.0 ± 2.2% (sensitivity: 60.1 ± 2.7%, specificity: 63.8 ± 2.0%) in the visual paradigm and 61.7 ± 1.5% (sensitivity: 62.5 ± 1.8%, specificity 60.9 ± 1.6%) in the auditory paradigm. In a one-way analysis of accuracy, it was found that accuracy in passive and breathing modes was significantly lower than that in active mode, where there was still a large difference between breathing and active modes. Besides, the difference in sensitivity and specificity was displayed in Figure 5.

**Table 5:** Classification results of EEG with different types of stimuli.

Paradigm	Classification samples	Performance	Active	Passive	Breathing	One-way ANOVA	P1	P2
Visual	Standard and deviant stimuli	Accuracy	75.5 ± 2.5%	63.8 ± 2.9%	55.6 ± 2.8%	**F(2, 57) = 13.49, *p* < 0.001, *η*2 = 0.32**	***p* = 0.011**	***p* < 0.001**
		Sensitivity	90.9 ± 0.8%	85.6 ± 1.2%	82.1 ± 1.4%	***F*(2, 57) = 14.17, *p* < 0.001, *η*2 = 0.33**	***p* = 0.006**	***p* < 0.001**
		Specificity	60.1 ± 4.1%	42.0 ± 4.6%	29.0 ± 4.3%	**F(2, 57) = 12.78, p < 0.001, *η*2 = 0.31**	***p* = 0.015**	***p* < 0.001**
	Correct and incorrect novel stimuli	Accuracy	62.0 ± 2.2%	51.9 ± 6.7%	51.2 ± 3.3%	**F(2, 57) = 14.31, *p* < 0.001, *η*2 = 0.33**	***p* < 0.001**	***p* < 0.001**
		Sensitivity	60.1 ± 2.7%	53.4 ± 2.2%	51.0 ± 1.5%	**F(2, 57) = 4.61, *p* = 0.014, *η*2 = 0.14**	*p* = 0.110	***p* = 0.014**
		Specificity	63.8 ± 2.0%	50.4 ± 1.9%	51.5 ± 1.4%	**F(2, 57) = 17.22, *p* < 0.001, *η*2 = 0.38**	***p* < 0.001**	***p* < 0.001**
Auditory	Standard and deviant stimuli	Accuracy	66.5 ± 1.5%	62.7 ± 1.4%	59.8 ± 1.5%	**F(2, 57) = 5.24, *p* = 0.008, *η*2 = 0.16**	*p* = 0.232	***p* = 0.006**
		Sensitivity	87.5 ± 0.7%	86.3 ± 0.6%	85.1 ± 0.8%	F(2, 57) = 2.58, *p* = 0.084, *η*2 = 0.08	*p* = 0.943	*p* = 0.081
		Specificity	45.5 ± 2.4%	39.1 ± 2.3%	34.4 ± 2.3%	**F(2, 57) = 5.47, *p* = 0.007, *η*2 = 0.16**	*p* = 0.182	***p* = 0.005**
	Correct and incorrect novel stimuli	Accuracy	61.7 ± 1.5%	57.0 ± 1.4%	53.9 ± 1.0%	**F(2, 57) = 8.92, *p* < 0.001, *η*2 = 0.24**	***p* = 0.042**	***p* < 0.001**
		Sensitivity	62.5 ± 1.8%	56.4 ± 2.1%	53.7 ± 1.5%	**F(2, 57) = 6.04, *p* = 0.004, *η*2 = 0.17**	*p* = 0.063	*p* = 0.483
		Specificity	60.9 ± 1.6%	57.6 ± 1.8%	54.0 ± 1.3%	**F(2, 57) = 4.72, *p* = 0.013, *η*2 = 0.14**	*p* = 0.431	***p* = 0.010**

P1 represents the results of the *post-hoc* analysis of the active and passive modes, P2 represents the results of the *post-hoc* analysis of the active and breathing modes. *p*-values < 0.05 are bolded.

**Figure 6:**
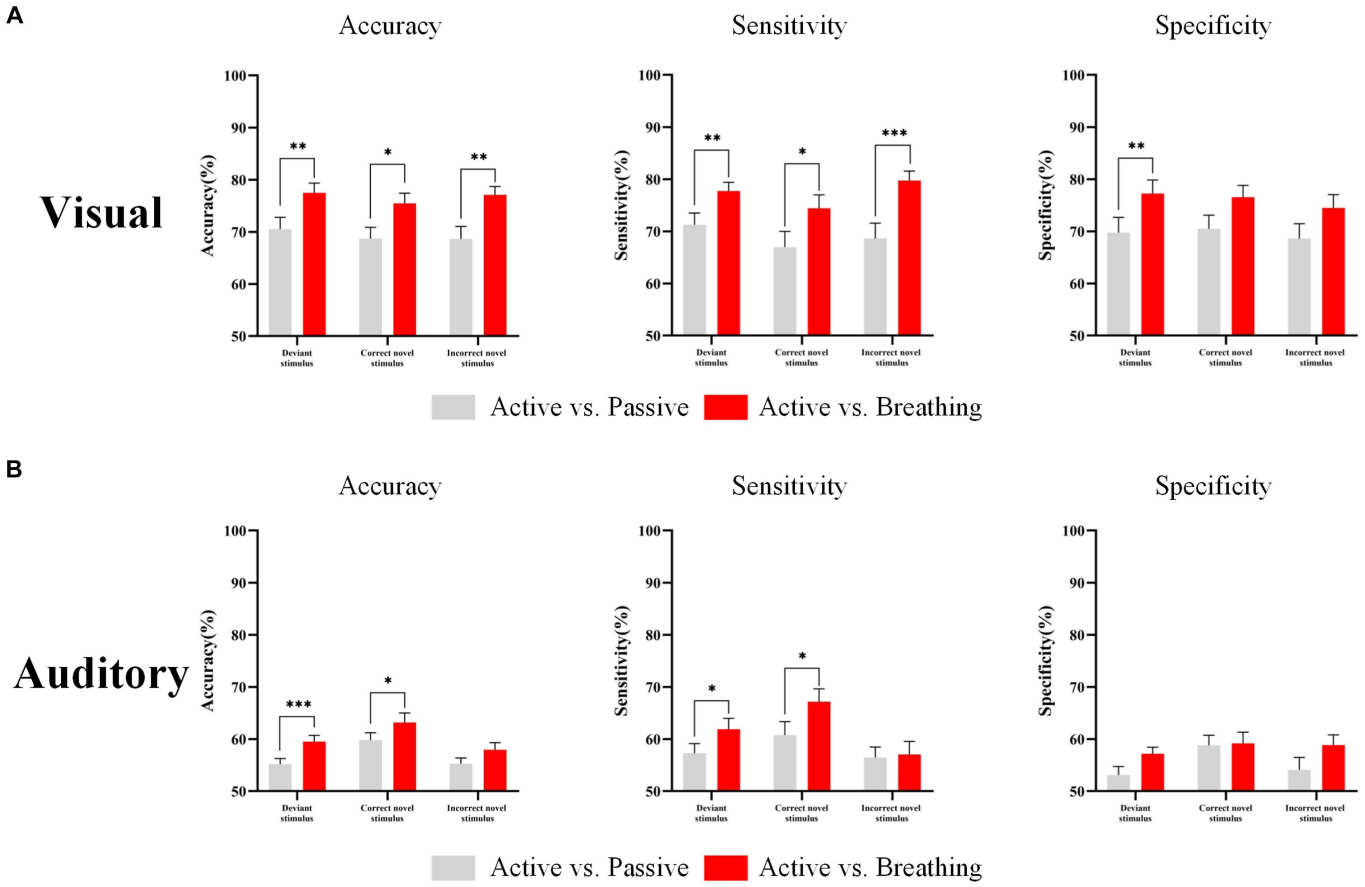
EEG response classification performance in different modes. Panel **(A)** shows the classification accuracy, sensitivity and specificity of the EEG responses in different modes under the visual paradigm. Panel **(B)** shows the classification accuracy, sensitivity and specificity of the EEG responses in different modes under the auditory paradigm. In this figure, ‘*’ denotes *p*  <  0.05, ‘**’ denotes *p*  <  0.01, ‘***’ denotes *p*  <  0.001.

The study classified the EEG responses of deviant stimuli, and novel stimuli in different modes and calculated the accuracy, sensitivity, and specificity. In Table 6, we can observe that under the visual paradigm, the classification accuracy of EEG responses in distinguishing the active and breathing modes is significantly higher than the classification accuracy of EEGs in distinguishing the active and passive modes. This implies that the EEG in active and breathing modes have better distinguishability. In the auditory paradigm, the active and respiratory modes had significantly better discriminability for all stimuli except for the incorrect novel stimuli. Besides, the difference in sensitivity and specificity was displayed in Figure 6. Overall, for conscious subjects, the SVM classifier was able to effectively discriminate between EEG responses in active and breathing modes.

**Table 6:** Classification results of EEG in different modes.

Paradigm	Stimulation	Performance	Active and passive modes	Active and breathing modes	Paired *t*-test results
Visual	Deviant stimulus	Accuracy	70.5 ± 2.3%	77.5 ± 1.9%	***p* = 0.003, *t* = −3.47**
		Sensitivity	71.3 ± 2.3%	77.7 ± 1.7%	***p* = 0.006, *t* = −3.08**
		Specificity	69.8 ± 2.9%	77.3 ± 2.6%	***p* = 0.006, *t* = −3.06**
	Correct novel stimulus	Accuracy	68.7 ± 2.1%	75.5 ± 1.9%	**p** = **0.010**, **t** = **−2.85**
		Sensitivity	67.0 ± 3.1%	74.4 ± 2.6%	***p* = 0.012, *t* = −2.76**
		Specificity	70.5 ± 2.6%	76.5 ± 2.3%	*p* = 0.059, *t* = −2.00
	Incorrect novel stimulus	Accuracy	68.6 ± 2.4%	77.1 ± 1.6%	***p* = 0.001, *t* = −3.87**
		Sensitivity	68.6 ± 2.9%	79.8 ± 1.8%	***p* < 0.001, *t* = −4.02**
		Specificity	68.6 ± 2.8%	74.5 ± 2.6%	*p* = 0.064, *t* = −1.96
Auditory	Deviant stimulus	Accuracy	55.2 ± 1.1%	59.5 ± 1.2%	***p* < 0.001, *t* = −4.08**
		Sensitivity	57.3 ± 1.9%	61.9 ± 2.1%	***p* = 0.025, *t* = −2.44**
		Specificity	53.1 ± 1.6%	57.2 ± 1.2%	*p* = 0.058, t = −2.02
	Correct novel stimulus	Accuracy	59.8 ± 1.4%	63.2 ± 1.8%	***p* = 0.019, *t* = −2.55**
		Sensitivity	60.8 ± 2.6%	67.2 ± 2.5%	***p* = 0.003, *t* = −2.36**
		Specificity	58.8 ± 2.0%	59.2 ± 2.2%	*p* = 0.868, *t* = −0.17
	Incorrect novel stimulus	Accuracy	55.3 ± 1.1%	58.0 ± 1.4%	*p* = 0.089, *t* = −1.79
		Sensitivity	56.5 ± 2.0%	57.1 ± 2.5%	*p* = 0.820, *t* = −0.23
		Specificity	54.1 ± 2.4%	58.9 ± 1.9%	*p* = 0.126, *t* = −1.60

*p*-values < 0.05 are bolded.

For the active and breathing paradigms, we used increasing the number of trials averaged and calculated the accuracy again, as shown in Table 7. By increasing the number of trials averaged, the classification accuracy of different stimuli in the active mode was increased, while the accuracy in the breathing paradigm did not change much. Meanwhile for distinguishing the EEG responses under the same stimulus in different modes, increasing the number of trial averages was also able to improve the classification accuracy, even reaching 90% in the visual paradigm. It is certain that by increasing the number of trials averaged, some of the non-locking noise in the EEG is attenuated and the potential features are more obvious, which is beneficial to the classification of the EEG. However, for the breathing mode or for all modes in subjects without consciousness, increasing the number of trial averages is not effective in improving the accuracy.

**Table 7:** Changes in the accuracy of increasing the number of trials averaged.

Paradigm	Mode	Stimulation	Num_avg = 1	Num_avg = 2	Num_avg = 3	Num_avg = 4	Num_avg = 5	Change in accuracy
Visual	Active	Standard and deviant stimuli	75.5%	79.3%	80.9%	81.9%	82.6%	**7.1%**
		Correct and incorrect novel stimuli	62.0%	65.2%	67.0%	68.2%	70.0%	**8.0%**
	Breathing	Standard and deviant stimuli	55.6%	56.2%	56.7%	57.1%	57.1%	1.5%
		Correct and incorrect novel stimuli	51.2%	51.8%	51.0%	52.3%	50.8%	−0.4%
	Active and breathing	Deviant stimulus	77.5%	83.6%	87.2%	89.1%	90.9%	**13.4%**
		Correct novel stimulus	75.5%	80.7%	83.7%	86.8%	88.2%	**12.7%**
		Incorrect novel stimulus	77.1%	83.8%	86.9%	89.0%	90.4%	**13.3%**
Auditory	Active	Standard and deviant stimuli	66.5%	69.4%	70.4%	71.1%	71.3%	4.8%
		Correct and incorrect novel stimuli	61.7%	64.8%	68.1%	69.1%	69.7%	**8.0%**
	Breathing	Standard and deviant stimuli	59.8%	60.8%	61.0%	61.0%	60.9%	1.1%
		Correct and incorrect novel stimuli	53.9%	55.7%	56.0%	57.0%	58.0%	4.1%
	Active and breathing	Deviant stimulus	59.5%	62.9%	65.2%	66.6%	68.2%	**8.7%**
		Correct novel stimulus	63.2%	66.2%	68.1%	70.2%	70.9%	**7.7%**
		Incorrect novel stimulus	58.0%	60.1%	60.9%	61.0%	62.5%	4.5%

Num_Avg is the number of trials averaged. Accuracy change values greater than 5% are bolded.

Three expected metrics for evaluating consciousness were identified through classification: (1) the accuracy of classifying EEG responses to different stimuli in the active mode. (2) The accuracy of classifying EEG responses distinguishing between the active and breathing modes for each type of stimulus. (3) The change in accuracy when increasing the average number of trials.

## 4 Discussion

In order to explore sensitive and accurate methods of consciousness detection which can be applied to clinical patients with DOC, We designed a new paradigm based on a multi-stage cognitive task capable of inducing a series of ERPs responding to different contents of consciousness as well as ERD/ERS phenomena. The EEG responses of 20 subjects under the paradigm were analyzed, and the time-domain and time-frequency-domain characteristics of the EEG responses evoked by standard stimuli, deviant stimuli, and novel stimuli in the active mode, the passive mode, and the breathing mode were compared. The extent of differences in EEG response under different conditions was quantified using the machine learning method.

We found that the novel paradigm was able to induce a series of ERPs or ERD/ERS changes, which reflect consciousness contents such as semantic processing and attentional engagement. In the active mode of visual and auditory stimulation, the brain was more activated, manifested as higher ERP amplitudes or more pronounced ERD/ERS phenomena. In the breathing mode, the level of brain activation was lower, and there were more significant differences in time and frequency domain between the breathing mode and the active mode compared to the passive mode. Combining machine learning to quantify the differences in EEG response under different conditions will enable sensitive and accurate consciousness detection in healthy subjects. It will lay the foundation for future application to consciousness detection in clinical patients with DOC.

### 4.1 The EEG characteristics that prove the existence of consciousness

P300 is considered to be an ERP component that is closely related to brain cognitive function and is one of the most commonly used components for consciousness detection (Annen et al., 2020). In the active mode of our designed consciousness detection paradigm, we observed a significant P300 component in the subjects’ EEG responses. In the passive mode, the P300 amplitude of the subjects was reduced, while it was further reduced in the breathing mode. Most studies showed that P300 tended to be detectable in healthy persons and MCS patients, while it was not found in most UWS patients. In addition, it has also been shown that compared to the passive mode of receiving stimuli, MCS patients tended to have a more excellent P300 component under the active mode of counting, whereas it was not observed in UWS patients (Hauger et al., 2015). However, in our study, we found that the P300 component is also present in passive mode, which would lead to misdiagnosis of some conscious patients who complete active and passive tasks as required.

CNV is associated with anticipation of upcoming stimuli. In the paradigm we designed, the appearance of deviant stimuli triggers the brain’s anticipation of novel stimuli. A significant CNV component was detected in the active mode of the auditory paradigm. In the auditory paradigm, we found a significant difference in CNV amplitude between the active and breathing modes. It has been found that CNV could be observed in EEG response of some patients with DOC and healthy subjects, and those patients with impaired consciousness who had CNV were more likely to detect abnormalities in stimulus patterns and are more likely to be conscious. The presence of CNV may mark the preservation of brain functions essential for consciousness in the forebrain (Sergent et al., 2017), implying partial preservation of functions in the anterior cingulate cortex and the prefrontal cortex, the two critical regions that carry out conscious processing. Therefore, focusing on the differences in the amplitude of the CNV under different conditions has essential implications for consciousness detection.

In the novel stimuli, we mainly focused on N400 and P600. N400 and P600, as endogenous components of ERP, usually appear in the case of speech and pictures contrary to expectation and respond to the brain’s cognitive processing of information (Erlbeck et al., 2017). In our study, there were significant N400 and P600 components in the active mode of the visual paradigm. There was a significant difference in P600 amplitude between the active mode and the breathing mode. There was a significant N400 in the active mode of the auditory paradigm, which was significantly different from the breathing mode. It has been found that N400 is observed in healthy persons (Rohaut et al., 2015), some MCS and *VS* patients, while P600 is present only in healthy persons and MCS patients. This is due to the fact that semantic processing in the brain is divided into two stages (Rohaut and Naccache, 2017). The N400 component represents the early, localized (verbal semantic network), unconscious stage of semantic processing. The late P600 component, which persists under the whole-brain scope, represents the stage of consciously acquiring semantic information and processing it. In these studies, common word pairs were presented to the subjects as stimuli, and depending on the actual experience of the subjects, not all of them were effectively evoked by the N400, P600, and a more effective stimulus was needed.

In contrast to these single-potential studies, our study designed a method of consciousness detection that includes multiple cognitive stages, which can efficiently evoke EEG characteristics related to consciousness, which will provide more analyzable characteristics for future application in patients. In addition, the subjects’ stimuli used in the study were more effective in evoking potentials than common stimuli such as tones and common word pairs.

Since the distribution of spectral oscillations in the brain plays an important role in cognitive processing with conscious involvement (Yordanova et al., 2020), we did not limit our analysis to the temporal domain and characterized the temporal-frequency domain. In the theta rhythm, which is related to the encoding and retrieval of events in memory (Jensen and Tesche, 2002), as shown by the results of our experiments, subjects with consciousness show different levels of activation during information and memory processing due to differences in stimulus type and task mode. In the alpha rhythm, which responds to the degree of neuronal processing activity(Yang et al., 2020), we observed a more pronounced alpha phenomenon in the active mode, implying that more brain resources were devoted to the active mode. These time-domain features along with time-frequency domain features are what we need to focus on in consciousness detection.

### 4.2 Active and breathing modes

The active and passive modes are the most frequently used in studies of consciousness detection. In the passive mode, subjects are often not required to respond, and because brain activity is different in patients with different states of consciousness, it is possible to determine the level of consciousness through features such as entropy, power spectrum, and complexity (Altintop et al., 2022, 2023). However it is not sufficient to use the passive mode alone, in fact, even if the brain in the passive mode is able to process a given stimulus continuously, it is not possible to distinguish whether this is a voluntary or automatic cognitive process, and therefore whether this is a conscious or unconscious brain processing (Schnakers et al., 2008). Thus, a comparison of the EEG responses in the two modes is necessary. The active mode requires subjects to respond to a specific stimulus, and comparing the EEG responses in these two modes enables us to understand whether the brain processes the stimulus consciously or unconsciously. At this point a new problem arises, if the difference between the active and passive paradigms is not large enough (Morlet et al., 2017), then we cannot well judge whether the brain’s response is consciously involved or not. In this study, breathing mode was introduced instead of passive mode. Breathing mode is a simple way to reduce the processing of external stimuli by the brain, thereby decreasing the event-related potential amplitude or the degree of brain functional connectivity (Atchley et al., 2016; Zhang et al., 2019). The results of this study show that the breathing mode showed significant differences from the active mode, allowing us to detect consciousness more sensitively.

### 4.3 Consciousness detection based on machine learning

In this study, we further used classification accuracy to quantify the degree of difference in EEG response between stimuli and between modes in the visual and auditory paradigms based on analysis of multi-potential features.

**Figure 7:**
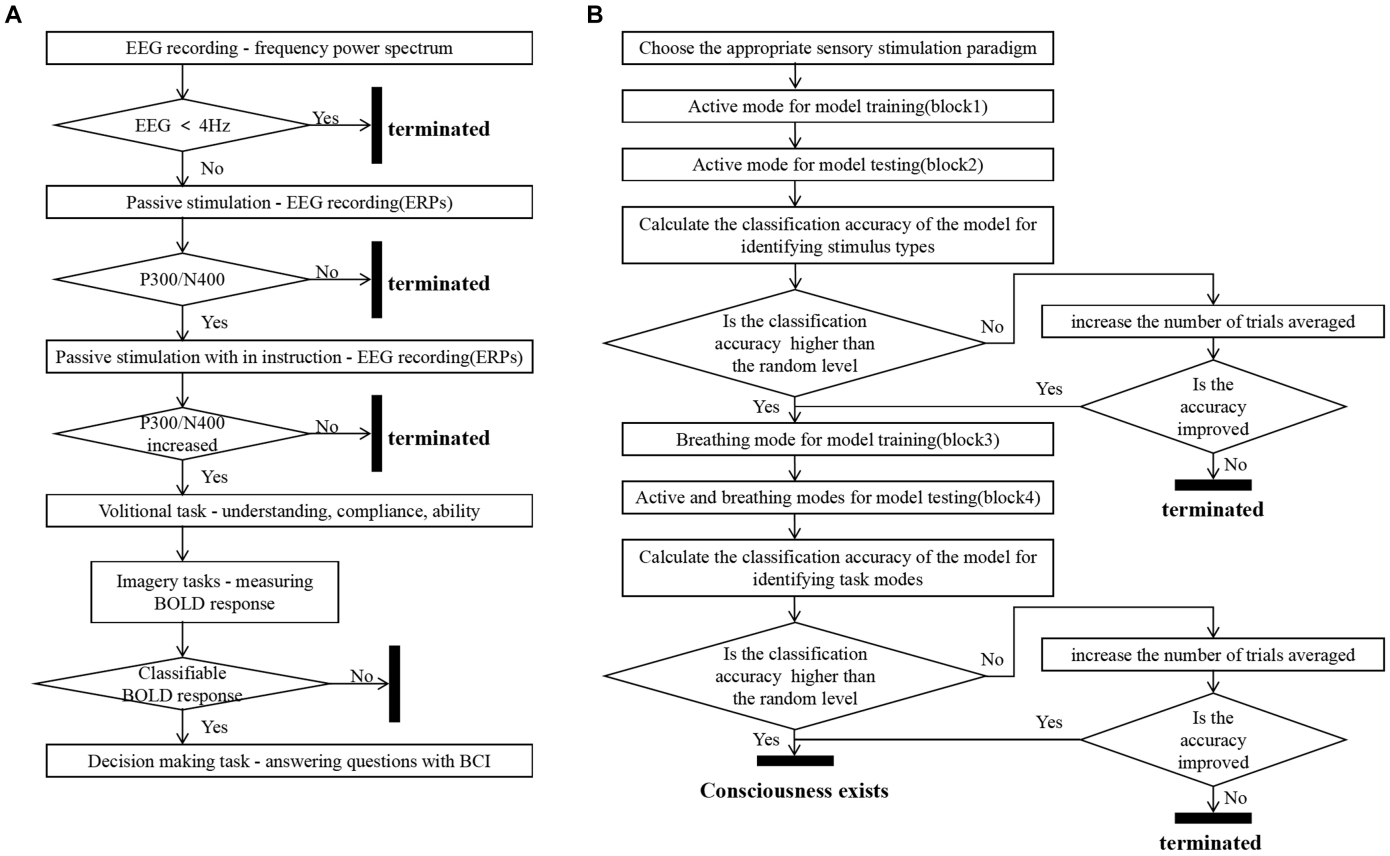
Consciousness detection process. Panel **(A)** shows Kuebler’s consciousness detection process (Kuebler and Kotchoubey, 2007). Panel **(B)** shows the consciousness detection process proposed in this study.

The study preliminarily designed a machine-learning-based consciousness detection process based on three metrics: the accuracy of classifying EEG responses to different stimuli in the active mode, the accuracy of classifying EEG responses distinguishing between the active and breathing modes for each type of stimulus, and the change in accuracy when increasing the average number of trials. The overall consciousness detection process was divided into two rounds of data collection training and two rounds of data collection testing, as shown in Figure 7. We illustrate the advantageousness and the specific procedure of our proposed method by comparing it with the process of consciousness detection proposed by Kuebler and Kotchoubey (2007). They classified patients with disorders of consciousness into three categories by means of step 2 passive stimulation and step 3 passive stimulation with in instruction (active mode):

1. Subjects without the presence of the P300/N400 in passive mode, which were considered by them to be unconscious. In fact, this ignores some of the subjects with potentials present in the active mode (Hauger et al., 2015). In our approach, the first concern is the classification accuracy of the different stimuli in active mode. When the accuracy is not higher than the randomized level, the number of trials averaged is increased to reduce the interference of noise on the EEG and to reconfirm that their EEG responses in the active mode are distinguishable (Ortner et al., 2017). When the classification accuracy of different stimuli in active mode is higher than the random level, the patient will proceed to the next step of consciousness detection.
2. Subjects with P300/N400 in the passive mode, but not significantly different from the active mode, are considered to be potentially conscious. However, the reason for the non-significant difference may not be that the subject failed to complete the active task, but rather that the difference between the passive and active modes is inherently small. In our approach, we determined whether there were differences between the modes by analyzing the accuracy of EEG responses distinguishing between the active and breathing modes for each type of stimulus as well as the change in accuracy when increasing the average number of trials, which would be more promising to be able to detect the presence of consciousness.
3. Subjects who had a P300/N400 in the passive mode, which was significantly different from the active mode, were considered to be conscious. In our approach, such subjects will demonstrate higher levels of the accuracy of classifying EEG responses to different stimuli in the active mode and the accuracy of distinguishing EEG responses between active and breathing modes for each type of stimulus than randomized levels, as well as an increase in accuracy as the average number of trials increases.

With our method of consciousness detection, the consciousness of more patients who are actually in the MCS state is expected to be detected, thus enabling timely and correct treatment to be given to the patients. In the future, the feasibility of this method will be verified on patients with impaired consciousness.

### 4.4 Limitations

In this study, we designed a novel consciousness detection paradigm and demonstrated the superiority of the breathing mode, and preliminarily proposed a machine learning-based consciousness detection method. There are certain shortcomings that need to be further corrected. First, this study did not collect data from clinical patients and included only 20 conscious, healthy subjects. We determined the presence or absence of consciousness through a combination of higher cognitive functions related to consciousness, and a positive result after the detection of consciousness tends to indicate the presence of consciousness in subjects with a series of higher cognitive processing abilities. However, a negative result does not necessarily mean that the subject is not conscious. Subjects who are conscious but lack higher cognitive processing abilities, such as attention and memory, may also yield negative results. Therefore, in further studies, we need to include DOC patients to further analyze the EEG response in the absence of consciousness, making the negative results effectively represent the absence of consciousness rather than the absence of higher cognitive abilities. Second, the currently proposed consciousness detection method requires two rounds of training sample collection, and patients with impaired consciousness may suffer from fatigue and poor experience. To solve this problem, optimizing the paradigm design or designing a cross-subject model are feasible approaches. Third, the use of EEG sample features in this paper does not take into account nonlinear features such as entropy and functional connectivity, resulting in the loss of some useful information. Meanwhile, some more advanced machine learning methods such as neural networks can be subsequently applied in our consciousness detection to improve the accuracy of consciousness detection.

In conclusion, in the future, we will collect experimental data from patients and continue to optimize our consciousness detection method based on the actual situation of our patients. At the same time, we will use some new machine learning techniques to improve the accuracy of our consciousness detection method. We believe that our proposed consciousness detection method that combines active and breathing modes will contribute to the research in the field of consciousness detection.

## 5 Conclusion

In this paper, we proposed a novel consciousness detection paradigm and a kind of multi-feature joint analysis method. In the consciousness detection paradigm, we designed a multi-stage cognitive task to induce ERPs and ERD/ERS phenomena representing different consciousness contents, such as P300, N400, CNV, and P600. A breathing mode was established to increase the sensitivity of consciousness detection that we verified to be superior to the passive mode. Based on this paradigm, we used a multi-feature joint analysis approach to comprehensively assess the brain’s level of consciousness and the accuracy of consciousness detection can be increased. The degree of EEG differences between conditions was quantified by machine learning. The experimental results suggested that multi-feature joint analysis of EEG responses in active and breathing modes was a more sensitive and accurate method of consciousness detection for healthy people. Future studies will continue to conduct experiments on patients with impaired consciousness to verify the reliability of our method. It is expected to provide accurate and sensitive consciousness detection for patients with DOC to reduce the probability of misdiagnosis. This will be of great significance for subsequent clinical rehabilitation treatment of patients.

## Data availability statement

The original data and materials presented in this article can be obtained from the corresponding authors upon request.

## Ethics statement

The studies involving humans were approved by the Bioethics Committee at Ningbo Institute of Materials Technology and Engineering, Chinese Academy of Sciences. The studies were conducted in accordance with the local legislation and institutional requirements. The participants provided their written informed consent to participate in this study. Written informed consent was obtained from the individual(s) for the publication of any potentially identifiable images or data included in this article.

## Author contributions

YY: Conceptualization, Formal analysis, Investigation, Methodology, Software, Visualization, Writing – original draft. YL: Data curation, Formal analysis, Methodology, Writing – review & editing. BY: Data curation, Investigation, Writing – review & editing. AY: Data curation, Software, Writing – original draft. HZ: Conceptualization, Funding acquisition, Methodology, Writing – review & editing. GZ: Conceptualization, Funding acquisition, Methodology, Project administration, Supervision, Writing – review & editing. JX: Conceptualization, Funding acquisition, Methodology, Project administration, Resources, Supervision, Writing – review & editing.
